# Supplementary material for: Enkephalin-mediated modulation of basal somatic sensitivity by regulatory T cells in mice
Source: eLife. 2024 Aug 7;13:RP91359. doi: 10.7554/eLife.91359 (PMC11305673; doi:10.7554/eLife.91359)
Supplement: Supplementary file 4 [file elife-91359-supp1.zip › Supplementary file 1.odt]

Supplementary File 1: Monoclonal antibodies used in the study of PenkCre x ROSA26tdTomato

| mAb or reagent (dye) | Provider | Catalog number | Clone | Dilution |
| --- | --- | --- | --- | --- |
| Anti-mouse F4/80 (AF647) | Biolegend | #123122 | BM8 | 1/400 |
| Anti-mouse CD62L (AF700) | BD Biosciences | #560517 | MEL-14 | 1/400 |
| Anti-mouse CD25 (APC Fire 750) | Biolegend | #102054 | PC61 | 1/100 |
| Anti-mouse CD4 (BUV 496) | BD Biosciences | #612952 | GK1.5 | 1/400 |
| Anti-mouse CD3 (BUV563) | BD Biosciences | #749277 | 145-2C11 | 1/400 |
| Anti-mouse CD69 (BUV661) | BD Biosciences | #741478 | H1.2F3 | 1/200 |
| Anti-mouse Nkp46 (BUV737) | BD Biosciences | #612805 | 29A1.4 | 1/200 |
| Anti-mouse CD8a (BUV805) | BD Biosciences | #612898 | 53-6.7 | 1/800 |
| Anti-mouse Ly6G (BV570) | Biolegend | #127629 | 1A8 | 1/200 |
| Anti-mouse CD11b (BV650) | Biolegend | #101259 | M1/70 | 1/1000 |
| Anti-mouse CD45 (NovaFluor Blue 660-120S) | ThermoFisher | #M005T02B08-A | 30-F11 | 1/200 |
| Anti-mouse CD11c (PE CF594) | BD Biosciences | #562454 | HL3 | 1/150 |
| Anti-mouse Ly6C (PE Cy7) | BD Biosciences | #560593 | AL-21 | 1/1600 |
| Anti-mouse CD44 (PerCP) | Biolegend | #103036 | IM7 | 1/200 |
| Anti-mouse CD19 (PerCP Cy5.5) | BD Biosciences | #551001 | 1D3 | 1/400 |
| Anti-mouse IA/IE (V500) | BD Biosciences | #562366 | M5/114.15.2 | 1/300 |
| Anti-mouse TCRγδ (BV421) | Biolegend | #109230 | GL3 | 1/200 |
| Anti-mouse Ki67 (AF532) | ThermoFisher | #58-5698-82 | SolA15 | 1/1600 |
| Anti-mCherry (Pacific Blue) | ThermoFisher | #M11238 | 16D7 | 1/50 |
| Anti-mouse Foxp3 (FITC) | ThermoFisher | #11-5773-82 | FJK-16s | 1/800 |
| Fixable viability dye (Live Dead Blue) | ThermoFisher | #L34961 |  | 1/2000 |
